# Admission-cohort differences in physical fitness, weight status, and fitness-standard failure among first-year students at a single vocational college in Shanxi Province, China

**DOI:** 10.3389/fpubh.2026.1850051

**Published:** 2026-08-10

**Authors:** Jing Hong

**Affiliations:** Department of Basic Education, Shanxi Railway Vocational and Technical College, Taiyuan, Shanxi, China

**Keywords:** China national student physical health standard, motor fitness, obesity, physical fitness, vocational college students, weight status

## Abstract

**Background:**

Physical fitness surveillance can identify students requiring early support, but evidence from vocational colleges remains limited.

**Methods:**

This repeated cross-sectional study analysed standardised physical fitness assessment records from 7,163 first-year students in three independent admission cohorts assessed in 2023, 2024, and 2025 at one vocational college in Shanxi Province, China. Official total scores and failure under China’s National Student Physical Health Standard were examined using descriptive analyses, quantile regression, logistic regression, and modified Poisson regression. Because BMI contributes directly to the official score, sensitivity analyses used a BMI-free motor-fitness score derived from the six performance-based components.

**Results:**

Official failure prevalence was 23.4% in 2023, 17.0% in 2024, and 34.8% in 2025. Compared with 2024, the 2025 cohort had a higher adjusted prevalence of official failure [adjusted prevalence ratio (aPR) 1.80, 95% confidence interval (CI) 1.64–1.99]. Median official scores differed modestly, whereas the adjusted 10th- and 25th-percentile scores were lower in 2025, indicating that the difference was concentrated in the lower part of the distribution. Weight-status associations were substantially attenuated after BMI removal, although overweight and obesity remained associated with low BMI-free motor-fitness performance (aPR 1.35, 95% CI 1.27–1.44, and aPR 1.64, 95% CI 1.55–1.74, respectively). Endurance failure was common in both sexes, and the high aggregate muscular strength/endurance failure prevalence was driven largely by male pull-up performance.

**Conclusion:**

Official fitness outcomes differed across the three admission cohorts, with the highest failure prevalence in 2025 reflecting lower-tail differences rather than a uniform decline. Weight-status disparities in official outcomes were partly attributable to the BMI component, although lower performance-based fitness persisted among students with overweight and obesity. These institution-specific findings support entry-level surveillance and targeted aerobic and muscular-endurance support but should not be generalised to vocational-college students across China.

## 1 Introduction

Physical fitness is a multidimensional construct reflecting cardiorespiratory endurance, muscular strength and endurance, flexibility, speed, and power. Higher physical fitness in young people and student populations has been associated with lower cardiometabolic risk, better musculoskeletal health, and more favourable long-term health outcomes (1–3). Physical fitness has also been associated with mental health among college students (4). Beyond representing current functional capacity, physical fitness is therefore an important marker of future health and a central outcome in sport and exercise science and public-health surveillance. Standardised assessment systems allow institutions to monitor fitness distributions, identify students below recommended performance thresholds, and examine disparities between demographic and educational groups. International surveillance studies have documented substantial changes in aerobic fitness and other fitness components among children, adolescents, and young adults, although the magnitude and direction of these changes have varied across populations and settings (5, 6).

In China, student physical fitness surveillance is implemented through China’s National Student Physical Health Standard, a nationally adopted framework used across educational stages (7). The standard combines anthropometric assessment with performance-based tests of endurance, muscular strength, speed, flexibility, and power to generate component scores and an official composite fitness score. National, provincial, and institution-level studies have demonstrated that physical fitness among Chinese college students varies across periods, regions, sexes, academic grades, majors, and institutional populations (8–12). Analyses of successive national surveys have identified long-term changes in college-student fitness and cardiovascular-risk indicators, while recent national data have also shown regional inequalities, increases in the prevalence of overweight and obesity, and non-linear associations between BMI and physical fitness (8, 12). These findings demonstrate the value of standardised surveillance but also indicate that national or long-term patterns should not automatically be assumed to apply uniformly to every institution. Repeated assessment of comparable first-year admission cohorts can therefore provide useful local surveillance evidence, provided that the findings are interpreted as differences between independent cohorts rather than as definitive national or secular trends.

Vocational college students represent an important but comparatively under-researched population within Chinese student-health research. Their educational experience may differ from that of students enrolled in general university programmes because vocational education frequently combines classroom instruction with practical training, work-placement preparation, and occupation-oriented skills development. Recent studies involving Chinese higher-vocational students have examined lifestyle-related weight change, sleep and mental well-being, and motivational influences on exercise adherence (13–15). These studies establish a relevant health and behavioural context for vocational students, but they do not directly examine standardised national physical-fitness outcomes. Evidence specifically addressing entry-level physical fitness, fitness-standard failure, and weight-related fitness disparities among first-year vocational college students therefore remains limited. Because some vocational programmes prepare students for occupations involving practical or physical demands, entry-level fitness may have implications for both long-term health and preparation for future study and employment.

Physical fitness and weight status may also be socially patterned. Chinese studies have linked physical activity and skeletal muscle mass with family affluence, parental education, and socioeconomic status, while national surveillance has identified regional inequalities in physical fitness and BMI (12, 16, 17). Such factors may shape prior educational opportunities and access to organised sport and physical education during the transition to vocational education. However, they were unavailable in the present dataset and were therefore treated as potential contextual influences rather than tested explanations for the observed admission-cohort or weight-status differences.

Associations between weight status and physical fitness have been reported consistently among Chinese university students. Large cross-sectional and multicentre studies have identified non-linear associations between BMI and performance across endurance, speed, strength, flexibility, and power-related components, with both low and high BMI frequently associated with poorer fitness (12, 18, 19). Body-composition research has further shown that higher fat mass is associated with poorer physical fitness and that students with normal BMI but excessive body fat may differ in skeletal muscle mass and functional performance from other normal-weight students (20, 21). These findings support examination of weight-related fitness disparities but also demonstrate that BMI does not directly distinguish fat mass from lean mass or fully represent functional capacity. Interpretation is additionally complicated under China’s National Student Physical Health Standard because BMI is used both to classify weight status and to contribute directly to the official total fitness score. Associations between weight status and the official total score or official failure may therefore partly reflect the design of the scoring system rather than BMI-independent differences in motor fitness.

Accordingly, this study had two objectives: (1) to describe differences in official national-standard physical fitness outcomes among three independent first-year admission cohorts assessed at one vocational college from 2023 to 2025; and (2) to examine weight-status differences in both official national-standard outcomes and BMI-free motor-fitness outcomes. The study’s main contribution was to determine whether differences in official failure prevalence reflected changes in median performance or differences in the lower tail of the score distribution, and whether weight-status associations persisted after removing the BMI component from the official score.

## 2 Materials and methods

### 2.1 Study design and participants

This repeated cross-sectional study used routinely collected physical fitness assessment records from Shanxi Railway Vocational and Technical College in Shanxi Province, China. Physical fitness assessments were conducted during the institution’s mandatory testing periods in 2023, 2024, and 2025. Each study year represented an independent admission cohort of first-year students who were admitted and assessed in the same calendar year. The three admission cohorts were therefore compared at a comparable educational stage. Because the same individuals were not followed over time, the analyses represent differences between independent first-year admission cohorts rather than longitudinal changes within individuals or population-level temporal trends.

All first-year students who completed China’s National Student Physical Health Standard assessment during the institutional testing period in 2023, 2024, or 2025 were eligible for inclusion. Records were included in the final analytic sample if data were available for sex, height, weight, weight status, official total physical fitness score, and total score grade. Records were excluded if key anthropometric or fitness assessment data were missing or if the record represented an incomplete assessment.

A total of 7,166 assessment records were initially available across the three cohorts. After data screening, three records from the 2023 cohort were excluded because height, weight, weight status, and component-level fitness test results were missing. No records were excluded from the 2024 or 2025 cohorts. The final analytic sample included 7,163 first-year students: 2,471 in 2023, 2,279 in 2024, and 2,413 in 2025. Date of birth was available in the administrative records. Because individual assessment dates were unavailable, age was calculated in decimal years using 1 March of the corresponding admission-cohort year as a common reference date within the January–March institutional testing period. The same method was applied consistently across all three cohorts. Differences in cohort size reflected the number of eligible first-year assessment records available in each year and did not represent attrition. Table 1 summarises participant inclusion and exclusion by admission cohort.

**Table 1:** Participant inclusion and exclusion by admission cohort.

Admission cohort	Initial records	Excluded records	Reason for exclusion	Final analytic sample
2023	2,474	3	Missing height, weight, weight status, and component-level fitness test data	2,471
2024	2,279	0	None	2,279
2025	2,413	0	None	2,413
Total	7,166	3	Incomplete assessment records	7,163

### 2.2 Physical fitness assessment procedures

Physical fitness was assessed in accordance with China’s National Student Physical Health Standard (7), issued by the Ministry of Education of China. This nationally implemented assessment integrates anthropometric measurements with performance-based tests designed to evaluate multiple components of health-related physical fitness, including cardiorespiratory endurance, muscular strength and endurance, flexibility, speed, and explosive power. Anthropometric measurements included height and body mass, which were measured using standardised equipment during the institutional testing period. Performance-based assessments included vital capacity, 50 m sprint, standing long jump, sit-and-reach test, endurance running, and muscular strength/endurance tests. Endurance was assessed using the 1,000 m run for male students and the 800 m run for female students, while muscular strength/endurance was assessed using pull-ups for male students and one-minute sit-ups for female students.

All tests were administered by trained institutional staff in accordance with standardised national protocols, and sex-specific testing procedures were applied where required. Assessments were conducted during the institutional physical fitness testing period from January to March in each assessment year. According to institutional records, the formal testing window, assessment protocol, scoring criteria, and equipment were unchanged across the 2023, 2024, and 2025 cohorts. However, detailed information on day-to-day testing conditions, student motivation during testing, and local implementation was not available for analysis. Raw test results were first converted into standardised component scores according to the sex-specific scoring tables defined in China’s National Student Physical Health Standard (2014 revision). These conversions transformed raw measurements recorded in different units, including BMI category, vital capacity, sprint time, sit-and-reach distance, standing long jump distance, endurance-run time, and muscular strength/endurance performance, into comparable component scores. For timed events such as the 50 m sprint and endurance run, faster performance corresponded to a higher component score, whereas for distance, repetition, and capacity-based tests, higher raw performance corresponded to a higher component score according to the national scoring tables.

The weighted base physical fitness score was calculated from the standardised component scores. Under the college-student scoring framework, BMI contributes 15% of the weighted base score, while the remaining 85% is derived from six performance-based components: vital capacity, 50 m sprint, sit-and-reach, standing long jump, muscular strength/endurance, and endurance running. Under the national standard, additional performance bonus points may be awarded for exceptional performance in endurance running and the sex-specific muscular strength/endurance test. In the institutional records, these bonus points were recorded separately and added to the weighted base score to produce the official total physical fitness score. Official failure was defined as an official total score below 60.0 points. Detailed sex-specific component-score conversion tables, BMI criteria, performance-bonus criteria, and classification thresholds from China’s National Student Physical Health Standard (2014 revision), as applied in this study, are provided in Supplementary Material S1. The component-score conversions and weights are summarised in Table 2.

**Table 2:** Component score conversion and weights used in the official total physical fitness score.

Component domain	Raw test or measurement	Component-score conversion	Contribution
Body composition	BMI category	BMI component score based on national BMI scoring criteria	15%
Functional capacity	Vital capacity	Raw vital-capacity value converted to standardised score using national sex-specific scoring table	15%
Speed	50 m sprint	Raw sprint time converted to standardised score using national sex-specific scoring table	20%
Flexibility	Sit-and-reach	Raw distance converted to standardised score using national sex-specific scoring table	10%
Explosive power	Standing long jump	Raw jump distance converted to standardised score using national sex-specific scoring table	10%
Muscular strength/endurance	Pull-ups for male students/One-minute sit-ups for female students	Raw repetitions converted to standardised score using national sex-specific scoring table	10%
Endurance	1,000 m run for male students/ 800 m run for female students	Raw endurance-run time converted to standardised score using national sex-specific scoring table	20%
Weighted base score	BMI and six performance-based components	Sum of weighted standardised component scores	Base score, with component weights totalling 100%
Performance bonus points	Endurance run and sex-specific muscular strength/endurance test	Additional points awarded according to the national criteria for exceptional performance	Added after calculation of the weighted base score
Official total physical fitness score	Weighted base score and recorded performance bonus points	Weighted base score + recorded performance bonus points	Final score used for official grading and failure classification

Raw test results were converted into standardised component scores according to China’s national student physical health standard (2014 revision). The weighted base score was calculated from the BMI component and six performance-based components. BMI contributed 15% of the weighted base score, while the performance-based components contributed 85%. Recorded bonus points for exceptional performance in endurance running and muscular strength/endurance were added after calculation of the weighted base score to produce the official total physical fitness score.

### 2.3 Weight status classification

Body mass index (BMI) was calculated as body mass in kilograms divided by height in metres squared. Weight status was categorised using the BMI scoring criteria for college students in China’s National Student Physical Health Standard (2014 revision), rather than international adult BMI cut-offs. The criteria used in this study are summarised in Table 3.

**Table 3:** BMI classification criteria and BMI component scores used for weight-status grouping.

Weight status	Male students, BMI (kg/m^2^)	Female students, BMI (kg/m^2^)	BMI component score
Normal weight	17.9–23.9	17.2–23.9	100
Underweight	≤17.8	≤17.1	80
Overweight	24.0–27.9	24.0–27.9	80
Obese	≥28.0	≥28.0	60

BMI was calculated as body mass in kilograms divided by height in metres squared. Criteria were b—ased on the BMI single-item scoring table for college students in China’s national student physical health standard (2014 revision) and were applied consistently across the 2023, 2024, and 2025 cohorts.

For male students, BMI was classified as normal weight at 17.9–23.9 kg/m^2^, underweight at ≤17.8 kg/m^2^, overweight at 24.0–27.9 kg/m^2^, and obese at ≥28.0 kg/m^2^. For female students, BMI was classified as normal weight at 17.2–23.9 kg/m^2^, underweight at ≤17.1 kg/m^2^, overweight at 24.0–27.9 kg/m^2^, and obese at ≥28.0 kg/m^2^. Because the BMI category determines the BMI component score and this component contributes 15% of the official total physical fitness score, BMI contributes directly to both the official total score and, potentially, the official failure classification for students near the passing threshold. Therefore, analyses involving official total score and official failure were interpreted as national-standard surveillance analyses rather than as BMI-independent motor-fitness analyses.

These sex-specific BMI classification criteria were applied consistently across all three admission cohorts. No additional age-specific or cohort-specific modifications were applied, as all participants were first-year vocational college students assessed under the same college-student standard. During data verification, recorded weight-status categories were checked against BMI calculated from measured height and body mass. Because BMI does not differentiate between fat mass and lean mass, weight status in this study was interpreted as a pragmatic surveillance indicator rather than a direct measure of adiposity.

### 2.4 Outcomes

The primary surveillance outcomes were the official total physical fitness score and official failure of China’s National Student Physical Health Standard. The official total score was calculated according to the national scoring framework by combining BMI, vital capacity, 50 m sprint, sit-and-reach, standing long jump, muscular strength/endurance, and endurance running components using nationally specified weights. Official failure was defined as a total physical fitness score below 60.0 points, corresponding to a non-passing grade under China’s National Student Physical Health Standard (7). The weighted base physical fitness score was calculated as:

$\begin{array}{l} \text{Weighted base score}=(\mathrm{BMI}\;\text{component score}\times 0.15)\\ +(\text{vital}-\text{capacity score}\times 0.15)+(50\;m\;\text{sprint score}\times 0.20)\\ +(\mathrm{sit}-\text{and}-\text{reach score}\times 0.10)\\ +(\text{standing}-\text{long}-\text{jump score}\times 0.10)\\ +(\text{muscular strength}/\text{endurance score}\times 0.10)\\ +(\text{endurance}-\mathrm{run}\;\text{score}\times 0.20) \end{array}$

The official total physical fitness score was calculated as:

Official total physical fitness score = weighted base score + recorded performance bonus points.

This formula shows that BMI contributes directly to the official total score through its 15% weighting. In the BMI component, normal-weight students received 100 points, underweight and overweight students received 80 points, and obese students received 60 points according to the national BMI scoring criteria. Consequently, before performance-based components were considered, the BMI contribution to the weighted base score was 15 points for normal-weight students, 12 points for underweight and overweight students, and 9 points for students with obesity. Because BMI contributes directly to the official total score, additional sensitivity outcomes were created to reduce potential circularity between weight status and total-score outcomes. A BMI-free motor-fitness score was calculated by removing the BMI component, rescaling the six weighted performance-based components to a 0–100 base scale, and retaining any recorded performance bonus points:

$\begin{array}{l} \mathrm{BMI}-\text{free motor}-\text{fitness score}=[(\text{vital}-\text{capacity score}\\ \times 0.15+50\;m\;\text{sprint score}\times 0.20+\mathrm{sit}-\text{and}-\text{reach score}\\ \times 0.10+\text{standing}-\text{long}-\text{jump score}\times 0.10+\text{muscular}\;\\ \;\;\;\;\text{strength}/\text{endurance score}\times 0.10+\text{endurance}-\\ \;\;\;\;\mathrm{run}\;\text{score}\times 0.20)/0.85]+\text{recorded performance}\;\\ \;\;\;\;\text{bonus points} \end{array}$

This calculation removed the direct contribution of BMI scoring while retaining performance-related bonus points. Because bonus points could increase the recalculated score above 100, the BMI-free score should be interpreted as a research sensitivity outcome rather than an official national-standard score. For male students, muscular strength/endurance was represented by pull-up score and endurance running by 1,000 m run score. For female students, muscular strength/endurance was represented by one-minute sit-up score and endurance running by 800 m run score. A BMI-free low motor-fitness outcome was defined as a BMI-free motor-fitness score below 60.0 points and used only as a sensitivity outcome; because it excluded BMI scoring while retaining performance bonus points, it was not interpreted as official failure under China’s National Student Physical Health Standard. The 60-point threshold was applied only as an analogous sensitivity cut-off and has not been validated as an official or clinical threshold for the BMI-free score. Component-level outcomes were also examined. Non-passing performance for each component was defined as a component score below 60.0 points after raw performance values were converted to standardised component scores using the sex-specific national scoring tables. Component-level failure reflects a non-passing grade for the specific test and does not necessarily indicate failure of the overall official national standard.

### 2.5 Statistical analysis

Participant characteristics, weight-status distribution, and physical fitness outcomes were summarised using descriptive statistics stratified by admission cohort and sex. Continuous variables were summarised using medians and interquartile ranges, with means and standard deviations also reported as secondary descriptive statistics. Age was summarised using the median, interquartile range, and range because the cohorts included a small number of younger and mature-age entrants. Age was entered as a continuous covariate in decimal years in all adjusted regression models. Categorical variables were summarised using frequencies and percentages. Missing data were assessed for date of birth, derived age, sex, height, weight, weight status, official total physical fitness score, total score grade, and each component-level fitness test. Missing data rates for key analytic variables are reported in Table 4.

**Table 4:** Missing data rates for key analytic variables before exclusion of incomplete records.

Variable	Expected, *N*	Missing, *n*	Missing, *n* (%)
Date of birth/derived age	7,166	0	0.00
Sex	7,166	0	0.00
Height	7,166	3	0.04
Weight	7,166	3	0.04
Weight status	7,166	3	0.04
Official total physical fitness score	7,166	0	0.00
Total score grade	7,166	0	0.00
Vital capacity	7,166	3	0.04
50 m sprint	7,166	3	0.04
Standing long jump	7,166	3	0.04
Sit-and-reach	7,166	3	0.04
Endurance run	7,166	3	0.04
Muscular strength/endurance test	7,166	3	0.04

Expected N refers to the number of records initially available before exclusion of incomplete records. Endurance run refers to the 1,000 m run for male students and the 800 m run for female students. Muscular strength/endurance test refers to pull-ups for male students and one-minute sit-ups for female students. Blank values for sex-specific tests that were not applicable under China’s National Student Physical Health Standard were not treated as missing data.

Three records from the 2023 cohort had missing height, weight, weight status, and component-level fitness test results and were excluded prior to analysis. No records were excluded from the 2024 or 2025 cohorts. Following these exclusions, no missing data were present for derived age, sex, weight status, official total physical fitness score, or total score grade in the final analytic sample. Because only three records (0.04% of the initially available records) had incomplete key anthropometric and component-level data, complete-case analysis was used and multiple imputation was not considered necessary.

Component-level failure prevalence was calculated separately for male and female students within each admission cohort because the endurance and muscular strength/endurance tests differed by sex and because male students represented more than 70% of each cohort. Percentages were calculated using the corresponding sex-specific admission-cohort sample as the denominator. Male endurance and muscular strength/endurance were represented by the 1,000 m run and pull-ups, respectively, whereas female endurance and muscular strength/endurance were represented by the 800 m run and one-minute sit-ups, respectively. Aggregate component-failure percentages were not interpreted as representing equivalent performance patterns among male and female students.

Data quality checks were conducted before analysis. Records were screened for missing key variables, duplicate entries, internally inconsistent values, and biologically implausible anthropometric values. One implausible birth year in the 2023 dataset was corrected from 2023 to 2003 after confirmation that it was a typographical error. BMI was recalculated from measured height and weight and cross-checked against the recorded weight-status category. Official total physical fitness scores and component scores were inspected using descriptive statistics and graphical checks. Values appearing as extreme outliers in boxplots, including very low official total scores, were reviewed against available component-level records. Records were excluded only when key anthropometric or fitness assessment data were missing or when the record represented an incomplete assessment. Verified low official total scores were retained because they reflected valid non-passing performance under the national scoring system rather than data-entry error.

The distribution of official total physical fitness scores was assessed using Shapiro–Wilk tests and visual inspection of histograms and Q–Q plots. Because official total physical fitness scores deviated from normality, unadjusted comparisons of official total physical fitness scores across admission cohorts and weight-status categories were conducted using Kruskal–Wallis tests. For adjusted analyses of official total physical fitness scores, median quantile regression models were used to estimate adjusted differences in median total scores. Models comparing admission cohorts were adjusted for age, sex, and weight status, while models comparing weight-status categories were adjusted for age, sex, and admission cohort. To further examine distributional differences across cohorts, dispersion and shape statistics were calculated, including standard deviation, coefficient of variation, interquartile range, skewness, and kurtosis. Variance heterogeneity across admission cohorts was assessed using the median-centred Levene’s test. Additional quantile regression models were fitted at the 10th and 25th percentiles to examine whether cohort differences were more pronounced in the lower tail of the official total-score distribution.

The same descriptive and regression analyses were repeated using the BMI-free motor-fitness score as a sensitivity outcome. Median BMI-free motor-fitness scores were summarised by admission cohort and weight-status category. Adjusted median differences in BMI-free motor-fitness score were estimated using quantile regression models adjusted for age, sex, and admission cohort. BMI-free low motor-fitness performance was also analysed as a binary sensitivity outcome. These analyses were used to assess whether weight-status differences persisted after removing the direct contribution of BMI scoring from the outcome. Recorded performance bonus points were retained in all continuous and binary BMI-free sensitivity analyses.

Associations with official failure of China’s National Student Physical Health Standard were first examined using logistic regression models, with results presented as odds ratios and 95% confidence intervals. Logistic regression was appropriate for the binary official failure outcome because normality of the official total physical fitness score is not an assumption of logistic regression. Weight status was entered into regression models as a categorical variable, with normal weight used as the reference category. The overall adjusted models included age, sex, admission cohort, and weight status. Admission cohort was modelled categorically, and no linear temporal trend was estimated. Additional pairwise comparisons were conducted using 2024 as the reference admission cohort to directly compare the 2025 cohort with the 2024 cohort.

Because official failure was a common outcome, odds ratios were not used as the sole measure of association. To improve interpretability, modified Poisson regression models with a log link and robust sandwich standard errors were additionally fitted to estimate adjusted prevalence ratios and 95% confidence intervals for official failure and BMI-free low motor-fitness performance. Adjusted predicted probabilities were also estimated using marginal standardisation, with each exposure category set in turn while retaining the observed distribution of the remaining covariates, including age. Logistic regression odds ratios were retained for comparability with the original analysis, but adjusted prevalence ratios and adjusted predicted probabilities were added to provide more directly interpretable relative and absolute estimates.

To assess the sensitivity of associations in the overall official-failure model to unmeasured confounding, E-values were calculated from the adjusted prevalence ratios and from the confidence limits closest to the null (22). The E-value represents the minimum strength of association, on the prevalence-ratio scale, that an unmeasured confounder would need to have with both the exposure and official failure, conditional on the measured covariates, to move the observed association to the null. E-values were not used to address the structural overlap between BMI-based weight status and the BMI-containing official outcome; that issue was evaluated separately using BMI-free motor-fitness sensitivity analyses.

Sex-stratified analyses were conducted because testing protocols differed by sex and male students represented more than 70% of each cohort. Sex-specific descriptive statistics were reported by admission cohort. Sex-stratified logistic regression and modified Poisson regression models were fitted separately for male and female students, adjusting for age, admission cohort, and weight status. A sex-by-weight-status interaction term was tested in the adjusted logistic regression model using a global Wald test. Statistical significance was evaluated using a two-sided *α* level of 0.05, with emphasis placed on effect sizes and confidence intervals rather than sole reliance on *p*-values. Because multiple admission-cohort and weight-status contrasts were reported, Benjamini–Hochberg false discovery rate correction was applied as a sensitivity analysis to selected pairwise contrasts reported in the main logistic regression model. Bonferroni correction was also examined as a more conservative sensitivity check. Descriptive tables were prepared using Microsoft Excel. Statistical analyses were conducted using Python 3.13.5 with pandas 2.2.3, SciPy 1.17.0, and statsmodels 0.14.6. Figures were generated using matplotlib 3.10.8.

### 2.6 Ethics considerations

This study involved retrospective secondary analysis of administrative physical fitness assessment records collected during routine institutional testing at Shanxi Railway Vocational and Technical College. The study used only existing records and involved no additional intervention or participant contact. The analytic dataset was de-identified before analysis and did not include names, student identification numbers, residential addresses, contact information, or other direct personal identifiers. Formal ethics-committee review was determined not to be required by the Shanxi Office for Education Sciences Planning because the study involved secondary analysis of existing de-identified administrative data. Written institutional authorisation to use the student physical fitness assessment data for research purposes was granted by Shanxi Railway Vocational and Technical College. Authorisation for the 2023 data was issued under institutional document Jin Tie Yuan Zi [2023] No. 42, and authorisation was also granted for the 2024 and 2025 data. The requirement for individual informed consent was waived by the Shanxi Office for Education Sciences Planning because the study involved no additional intervention or participant contact and used only de-identified existing records. Data were stored and analysed confidentially, and no participant could be identified from the reported results. The study was conducted in accordance with the Declaration of Helsinki.

## 3 Results

### 3.1 Participant characteristics and weight status

A total of 7,163 first-year vocational college students were included in the final analytic sample, comprising 2,471 students assessed in 2023, 2,279 in 2024, and 2,413 in 2025. Median age was 18.0 years in the 2023 cohort, 17.9 years in the 2024 cohort, and 18.0 years in the 2025 cohort. The corresponding age ranges were 15.3–43.5 years, 15.3–56.1 years, and 15.3–47.7 years, respectively, reflecting the inclusion of a small number of younger and mature-age entrants. Male students constituted the majority of participants in each cohort, accounting for 72.28% in 2023, 70.38% in 2024, and 74.76% in 2025. Descriptive characteristics of the study population, including age, anthropometric measures, sex distribution, weight-status categories, combined overweight/obesity prevalence, and official failure prevalence, are presented in Table 5.

**Table 5:** Participant characteristics and weight-status distribution by admission cohort at the study institution (2023–2025).

Characteristic	2023 (*n* = 2,471)	2024 (*n* = 2,279)	2025 (*n* = 2,413)
Male, *n* (%)	1,786 (72.28%)	1,604 (70.38%)	1,804 (74.76%)
Female, *n* (%)	685 (27.72%)	675 (29.62%)	609 (25.24%)
Age, median (IQR), years	18.0 (17.7–18.5)	17.9 (17.6–18.4)	18.0 (17.7–18.4)
Height, mean (SD), cm	170.47 (8.11)	171.64 (8.24)	172.11 (8.18)
Weight, mean (SD), kg	68.05 (16.62)	67.62 (15.58)	70.14 (17.04)
Underweight, *n* (%)	229 (9.27%)	198 (8.69%)	195 (8.08%)
Normal weight, *n* (%)	1,306 (52.85%)	1,304 (57.22%)	1,243 (51.51%)
Overweight, *n* (%)	495 (20.03%)	479 (21.02%)	528 (21.88%)
Obese, *n* (%)	441 (17.85%)	298 (13.08%)	447 (18.52%)
Overweight or obese, *n* (%)	936 (37.88%)	777 (34.09%)	975 (40.41%)
Male overweight/obese, *n* (%)	752 (42.11%)	601 (37.47%)	806 (44.68%)
Female overweight/obese, *n* (%)	184 (26.86%)	176 (26.07%)	169 (27.75%)
Official failure prevalence, *n* (%)	578 (23.39%)	388 (17.03%)	839 (34.77%)

Percentages were calculated using the relevant denominator within each admission cohort after exclusion of incomplete records. Overall weight-status and official failure-prevalence percentages were calculated using the final analytic sample for each admission cohort. Sex-stratified overweight/obesity percentages were calculated using the number of male or female students within the corresponding admission cohort as the denominator. “Overweight/obesity combined” was calculated by summing the number of students classified as overweight and obese; it is not a separate BMI category. Weight status was classified according to the BMI scoring criteria for college students specified in China’s National Student Physical Health Standard (2014 revision). BMI was calculated as body mass in kilograms divided by height in m^2^. Age was calculated using 1 March of the corresponding admission-cohort year as a common reference date and is reported as median and interquartile range.

Recorded performance bonus points were uncommon, occurring in 34 students in 2023, 20 students in 2024, and 90 students in 2025, and ranged from 1 to 10 points. Across all three cohorts, normal weight was the most prevalent weight category, comprising approximately half of the student population. The combined prevalence of overweight and obesity varied across cohorts, accounting for 37.88% in 2023, 34.09% in 2024, and 40.41% in 2025. The 2024 cohort had the lowest combined overweight/obesity prevalence among the three cohorts, while the highest prevalence was observed in 2025. The prevalence of underweight remained relatively stable, at 9.27% in 2023, 8.69% in 2024, and 8.08% in 2025. Sex-stratified results showed consistently higher combined overweight/obesity prevalence among male students than female students across all cohorts: 42.11% versus 26.86% in 2023, 37.47% versus 26.07% in 2024, and 44.68% versus 27.75% in 2025. The largest male–female difference was observed in 2025.

### 3.2 Differences in official Total physical fitness scores between admission cohorts

**Figure 1:**
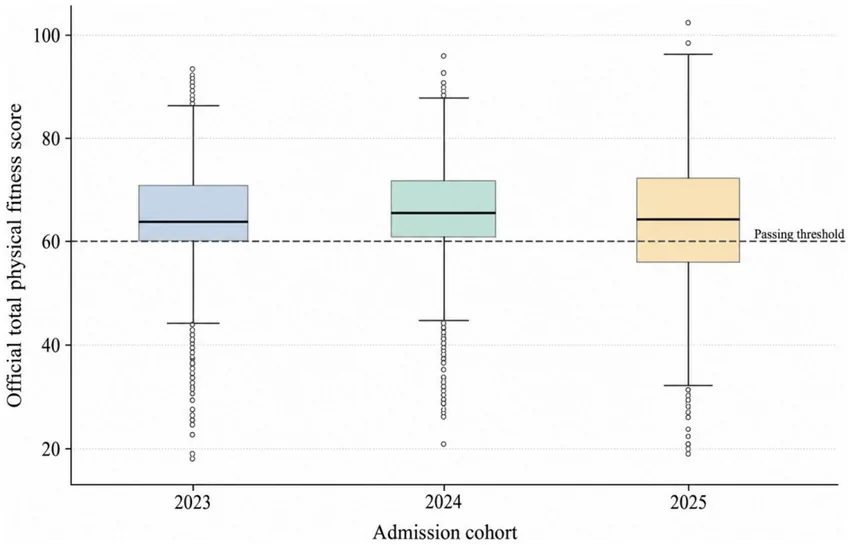
Distribution of official total physical fitness scores by admission cohort (2023–2025). Boxplots show median values, interquartile ranges, whiskers, and outlying observations for official total physical fitness scores in the study sample. Outlying observations were retained after data-quality checks when they reflected valid low performance rather than incomplete or erroneous records. The horizontal dashed line indicates the official passing threshold of 60 points.

The distribution of official total physical fitness scores varied across the three admission cohorts. Figure 1 presents the distribution of official total scores by admission cohort, showing differences in both central tendency and dispersion.

The median official total score increased modestly from 64.0 in 2023 to 65.6 in 2024, followed by a slight decrease to 64.6 in 2025. Mean official total scores showed a similar pattern, with values of 63.18 (SD 11.57) in 2023, 64.57 (SD 10.86) in 2024, and 64.24 (SD 12.95) in 2025. Although differences in central tendency across cohorts were small, the 2025 cohort showed greater dispersion than the 2023 and 2024 cohorts. The interquartile range was 10.65 in 2023, 10.90 in 2024, and 16.20 in 2025, while the coefficient of variation was 18.32, 16.81, and 20.15%, respectively. The median-centred Levene’s test indicated significant heterogeneity in official total-score variance across cohorts, W = 49.50, *p* < 0.001. Distributional characteristics, including skewness, kurtosis, and normality assessment results, are presented in Table 6.

**Table 6:** Distributional characteristics and normality assessment of official total physical fitness score by admission cohort.

Statistic	2023	2024	2025
n	2,471	2,279	2,413
Mean	63.18	64.57	64.24
SD	11.57	10.86	12.95
Median	64.0	65.6	64.6
IQR	10.65	10.90	16.20
CV (%)	18.32	16.81	20.15
Skewness	−0.67	−0.85	−0.28
Kurtosis	0.54	0.96	0.08
Shapiro–Wilk W	0.963	0.944	0.988
Shapiro–Wilk *p*-value	<0.001	<0.001	<0.001

CV, coefficient of variation; IQR, interquartile range; SD, standard deviation. CV was calculated as SD divided by the mean multiplied by 100. Skewness and kurtosis were calculated from the cleaned analytic dataset; kurtosis is reported as excess kurtosis. Shapiro–Wilk tests were used to assess normality within each admission cohort. All *p*-values were <0.001, indicating deviation from normality.

**Figure 2:**
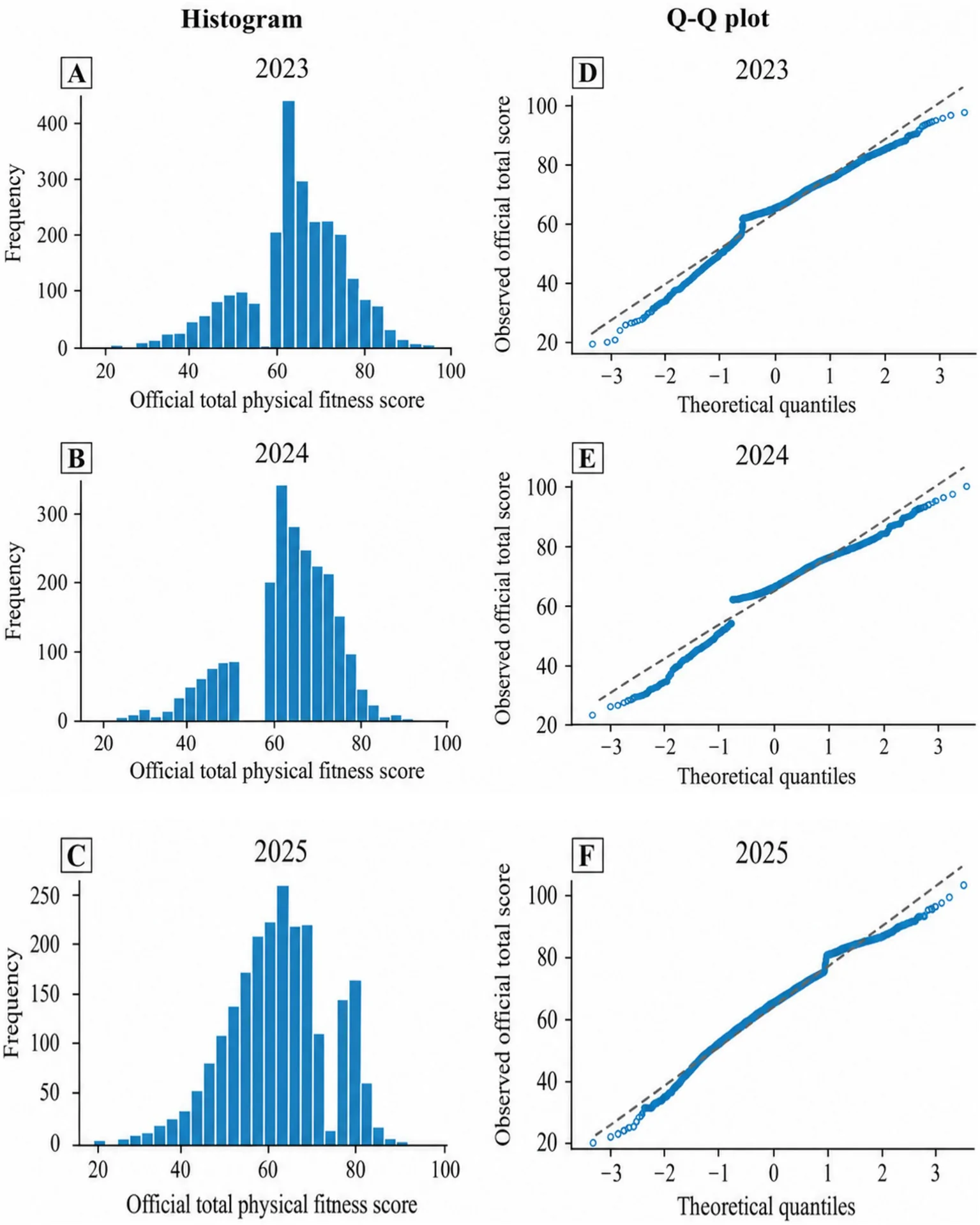
Histograms and Q–Q plots of official total physical fitness scores by admission cohort. Panels **(A–C)** show histograms of official total physical fitness scores for the 2023, 2024, and 2025 cohorts, respectively. Panels **(D–F)** show Q–Q plots comparing the observed official total-score distributions with the theoretical normal distribution for the 2023, 2024, and 2025 cohorts, respectively. The dashed reference line represents the expected normal distribution. Departures from the reference line, particularly in the lower and upper tails, indicate deviation from normality and support the Shapiro–Wilk test results.

Shapiro–Wilk tests indicated that official total physical fitness scores were not normally distributed in any cohort: 2023, W = 0.963, *p* < 0.001; 2024, W = 0.944, *p* < 0.001; and 2025, W = 0.988, *p* < 0.001. Visual inspection of histograms and Q–Q plots also indicated deviations from normality, particularly in the lower and upper tails of the distributions, and these diagnostic plots are provided in Figure 2. Accordingly, medians and interquartile ranges were emphasised when describing official total-score distributions, and non-parametric methods were used for unadjusted comparisons. The Kruskal–Wallis test indicated a statistically significant difference in official total-score distributions across admission cohorts, H(2) = 20.01, *p* < 0.001.

Lower-tail quantile regression further supported the distributional interpretation. In the direct comparison between 2025 and 2024, the adjusted 10th-percentile official total score was 3.10 points lower in 2025 (95% CI = −4.35 to −1.85, *p* < 0.001), and the adjusted 25th-percentile official total score was 1.24 points lower in 2025 (95% CI = −1.92 to −0.55, *p* < 0.001). In contrast, the adjusted median official total score did not differ significantly between the 2025 and 2024 cohorts (adjusted median difference = 0.36, 95% CI = −0.21 to 0.94, *p* = 0.214). These findings indicate that the higher official failure prevalence in 2025 was more consistent with lower-tail differences and greater dispersion than with a uniform downward shift in the official total-score distribution. Adjusted quantile regression results are presented in Table 7.

**Table 7:** Adjusted quantile regression estimates comparing 2025 with 2024.

Quantile	Adjusted difference, 2025 vs 2024	95% CI	*p*-value
10th percentile	−3.10	−4.35 to −1.85	<0.001
25th percentile	−1.24	−1.92 to −0.55	<0.001
50th percentile	0.36	−0.21 to 0.94	0.214

Models were adjusted for age, sex, and weight status. Age was entered as a continuous covariate in decimal years. Positive values indicate higher official total physical fitness scores in 2025 compared with 2024; negative values indicate lower official total physical fitness scores in 2025 compared with 2024.

In adjusted median quantile regression models controlling for age, sex, and weight status, the median official total fitness score did not differ significantly between the 2024 and 2023 cohorts (adjusted median difference = 0.35, 95% CI = −0.22 to 0.92, *p* = 0.232). The 2025 cohort had a slightly higher adjusted median score than the 2023 cohort (adjusted median difference = 0.71, 95% CI = 0.15 to 1.27, *p* = 0.013). Taken together, these findings indicate that the higher official failure prevalence in 2025 was not explained by a uniform decline in median official total fitness score but was more consistent with wider dispersion and a larger subgroup of low-performing students in that cohort.

### 3.3 Weight status, official Total score, and BMI-free motor-fitness score

**Figure 3:**
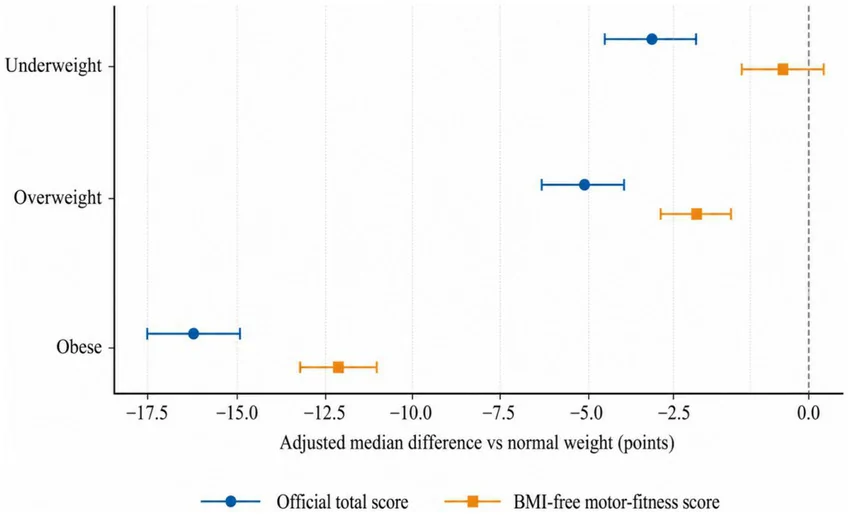
Adjusted median differences in official total physical fitness score and BMI-free motor-fitness score by weight status. Points indicate adjusted median differences compared with normal-weight students, and horizontal bars indicate 95% confidence intervals. Models were adjusted for age, sex, and admission cohort. The official total score includes the BMI component of China’s national student physical health standard. The BMI-free motor-fitness score removes the BMI component, rescales the six performance-based components to a 0–100 base scale, and retains recorded performance bonus points. The vertical dashed line indicates no difference from normal-weight students. The comparison shows attenuation of weight-status differences after BMI removal, although lower BMI-free motor-fitness performance remained evident among students with overweight and obesity.

Official total physical fitness scores differed across weight-status categories. To address the potential circularity caused by the inclusion of BMI in the official total score, Figure 3 compares adjusted median differences in the BMI-containing official total score with adjusted median differences in the BMI-free motor-fitness score. This comparison was used to assess whether weight-status differences persisted after removing the BMI component from the official scoring framework.

Adjusted median differences in official total score and BMI-free motor-fitness score are presented in Table 8.

**Table 8:** Adjusted median differences in official total score and BMI-free motor-fitness score by weight status.

Weight status	Official total score adjusted median difference vs normal weight	*p*-value	BMI-free motor-fitness adjusted median difference vs normal weight	*p*-value
Normal weight	Reference	—	Reference	—
Underweight	−3.76 (95% CI: −4.60 to −2.91)	<0.001	−0.88 (95% CI: −1.88 to 0.11)	0.082
Overweight	−5.76 (95% CI: −6.36 to −5.16)	<0.001	−3.25 (95% CI: −3.95 to −2.54)	<0.001
Obese	−16.28 (95% CI: −16.94 to −15.62)	<0.001	−12.06 (95% CI: −12.83 to −11.29)	<0.001

Estimates were derived from median quantile regression models adjusted for age, sex, and admission cohort. Age was entered as a continuous covariate in decimal years. Normal weight was used as the reference category. Official total score refers to the BMI-containing national-standard score. The BMI-free motor-fitness score removes the BMI component, rescales the six performance-based components to a 0–100 base scale, and retains recorded performance bonus points. CI, confidence interval.

In median quantile regression models adjusted for age, sex, and admission cohort, underweight, overweight, and obese students had lower official total scores than normal-weight students. Compared with normal-weight students, the adjusted median official total score was 3.76 points lower among underweight students, 5.76 points lower among overweight students, and 16.28 points lower among students with obesity. These official-score findings show graded differences across weight-status categories within the national-standard scoring framework. However, because BMI contributes directly to the official total score, these results should not be interpreted as fully independent motor-fitness differences.

Sensitivity analyses using the BMI-free motor-fitness score showed that weight-related differences were attenuated after removal of the BMI scoring component. The unadjusted median BMI-free motor-fitness score was 63.29 among normal-weight students, 62.21 among underweight students, 59.44 among overweight students, and 50.79 among students with obesity. In median quantile regression models adjusted for age, sex, and admission cohort, the BMI-free motor-fitness score was 0.88 points lower among underweight students than among normal-weight students, but this difference was not statistically significant (95% CI = −1.88 to 0.11, *p* = 0.082). In contrast, overweight and obesity remained associated with lower BMI-free motor-fitness performance. Compared with normal-weight students, the adjusted median BMI-free motor-fitness score was 3.25 points lower among overweight students and 12.06 points lower among students with obesity. These sensitivity findings indicate that the association between weight status and the official total physical fitness score was partly influenced by the inclusion of BMI in the national scoring system. However, lower performance-based motor fitness persisted among students with overweight and obesity after removal of the BMI component. Therefore, official total-score results should be interpreted as national-standard surveillance findings, while BMI-free motor-fitness results provide a more conservative assessment of performance-based fitness differences.

### 3.4 Failure of the national physical health standard

**Figure 4:**
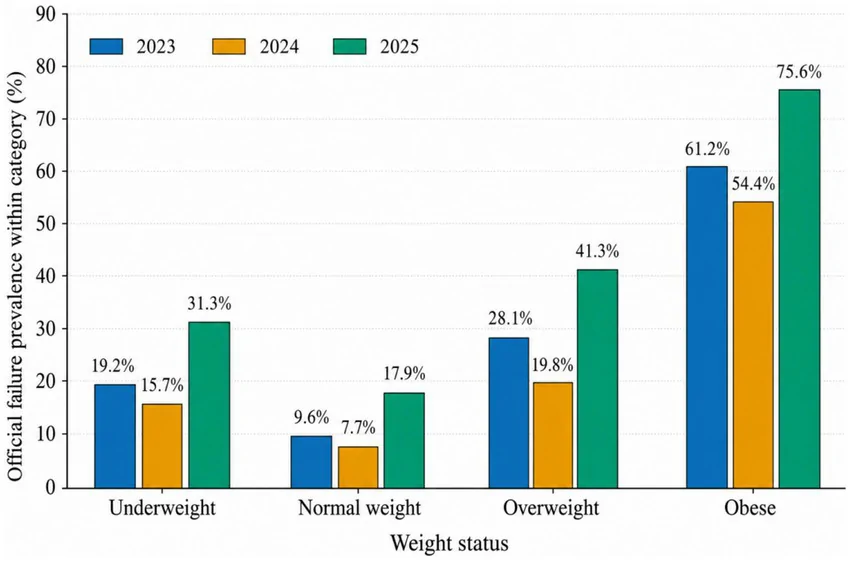
Prevalence of official failure of China’s National Student Physical Health Standard by admission cohort and weight status in the study sample. The bar chart shows the percentage of students within each weight-status category who failed to meet China’s National Student Physical Health Standard in 2023, 2024, and 2025. Percentages are calculated using all students within each weight-status category, with male and female students combined.

The prevalence of official failure of China’s National Student Physical Health Standard differed across the three independent admission cohorts and varied by weight-status category. Because official failure is based on the BMI-containing total score, weight-status differences in official failure were interpreted as national-standard surveillance findings rather than BMI-independent motor-fitness associations. Official failure prevalence differed across admission cohorts: 23.39% in 2023, 17.03% in 2024, and 34.77% in 2025. The 2024 cohort therefore represented the lowest observed failure prevalence across the three cohorts, while the 2025 cohort had the highest observed failure prevalence. Official failure prevalence was 17.74 percentage points higher in the 2025 cohort than in the 2024 cohort. Because the data were drawn from one institution and only three independent first-year admission cohorts, these results describe within-institution cohort differences rather than broader temporal or population-level trends. Prevalence of official failure by admission cohort and weight status is presented in Figure 4.

Official failure prevalence also showed a pronounced gradient across weight-status categories. In 2023, official failure prevalence was 19.2% among underweight students, 9.6% among normal-weight students, 28.1% among overweight students, and 61.2% among obese students. In 2024, the corresponding values were 15.7, 7.7, 19.8, and 54.4%. In 2025, these values increased to 31.3, 17.9, 41.3, and 75.6%, respectively. These descriptive findings indicate higher official failure prevalence across all weight-status categories in 2025 compared with 2024, while also showing consistently higher failure prevalence among students with overweight and obesity than among normal-weight students.

Sex-stratified descriptive characteristics are presented in Table 9. Male students had higher combined overweight/obesity prevalence than female students in each cohort. Crude official failure prevalence was also higher among male students across all three cohorts: 24.08% versus 21.61% in 2023, 19.20% versus 11.85% in 2024, and 37.53% versus 26.60% in 2025. Median official total fitness scores were lower among male students than female students in each cohort.

**Table 9:** Sex-stratified descriptive characteristics by admission cohort.

Admission cohort	Sex	*n*	Overweight/obese, *n* (%)	Official failure, *n* (%)	Median official total score (IQR)
2023	Male	1,786	752 (42.11%)	430 (24.08%)	63.6 (10.2)
2023	Female	685	184 (26.86%)	148 (21.61%)	66.0 (11.6)
2024	Male	1,604	601 (37.47%)	308 (19.20%)	64.2 (9.5)
2024	Female	675	176 (26.07%)	80 (11.85%)	69.6 (10.2)
2025	Male	1,804	806 (44.68%)	677 (37.53%)	63.4 (15.3)
2025	Female	609	169 (27.75%)	162 (26.60%)	68.7 (19.5)

Percentages for overweight/obesity and official failure were calculated within each sex-specific admission cohort subgroup. IQR, interquartile range.

Overall adjusted odds ratios and adjusted prevalence ratios for official failure are presented in Table 10. In the overall adjusted models, both the odds and prevalence of official failure were lower in the 2024 admission cohort than in the 2023 cohort and higher in the 2025 admission cohort than in the 2023 cohort. In the direct comparison between the two most recent cohorts, the 2025 cohort had higher odds and prevalence of official failure than the 2024 cohort after adjustment for age, sex, and weight status. Weight-status categories also differed in official failure after adjustment for age, sex, and admission cohort. Relative to normal-weight students, underweight students and students with overweight or obesity had higher odds and prevalence of official failure. Adjusted prevalence ratios were smaller than the corresponding odds ratios, as expected when the outcome is common.

**Table 10:** Overall adjusted odds ratios, adjusted prevalence ratios, and *E*-value sensitivity analyses for official failure of China’s national student physical health standard.

Predictor	Adjusted OR (95% CI)	aPR (95% CI)	*E*-value for point estimate; confidence-limit *E*-value
Admission cohort 2024 vs. 2023	0.73 (0.62–0.86)	0.80 (0.72–0.89)	1.80; 1.49
Admission cohort 2025 vs. 2023	1.95 (1.69–2.25)	1.45 (1.34–1.57)	2.26; 2.01
Admission cohort 2025 vs. 2024	2.67 (2.29–3.11)	1.80 (1.64–1.99)	3.01; 2.66
Underweight vs. normal weight	2.20 (1.77–2.74)	1.89 (1.59–2.24)	3.18; 2.56
Overweight vs. normal weight	3.24 (2.79–3.78)	2.52 (2.25–2.82)	4.47; 3.92
Obese vs. normal weight	14.49 (12.34–17.01)	5.31 (4.82–5.85)	10.08; 9.10
Female vs. male	0.96 (0.83–1.11)	0.97 (0.88–1.06)	1.22; 1.00

Adjusted OR, odds ratio; aPR, adjusted prevalence ratio; CI, confidence interval. ORs were estimated using logistic regression. aPRs were estimated using modified Poisson regression with a log link and robust sandwich standard errors. Overall models included age, sex, admission cohort, and weight status. Age was entered as a continuous covariate in decimal years but is not displayed as a focal predictor in the table. Normal weight, the 2023 admission cohort, and male sex were used as reference categories. The 2025-versus-2024 comparison was obtained using 2024 as the reference cohort. *E*-values were calculated from the aPRs; for associations below 1.00, reciprocal values were used. The confidence-limit E-value corresponds to the confidence bound closest to the null. An *E*-value of 1.00 is reported when the confidence interval includes the null.

*E*-value sensitivity analyses indicated that the magnitude of unmeasured confounding required to move the observed associations to the null varied across predictors. For the comparison between the 2025 and 2024 admission cohorts, the *E*-value was 3.01 for the adjusted prevalence ratio and 2.66 for the confidence limit closest to the null. For obesity compared with normal weight, the corresponding *E*-values were 10.08 and 9.10. Thus, under the assumptions of the E-value method, an unmeasured factor would need to be associated with both the exposure and official failure by prevalence ratios of at least these magnitudes, conditional on age and the other measured covariates, to move the respective estimates to the null. *E*-values for the remaining associations are presented in Table 10. These estimates provide a quantitative sensitivity assessment for unmeasured confounding but do not establish causality or address the structural inclusion of BMI in the official score.

Sex-stratified adjusted odds ratios and adjusted prevalence ratios are presented in Table 11. The 2025 admission cohort had higher odds and prevalence of official failure than the 2024 cohort among both male and female students after adjustment for age and weight status. Overweight and obesity were also associated with higher official failure in both sexes, although the magnitude of the weight-status associations differed. In particular, the association between obesity and official failure was larger among male students than among female students. The sex × weight-status interaction was statistically significant in the adjusted logistic regression model (global Wald test: *χ*^2^ = 22.65, df = 3, *p* < 0.001), indicating that the association between weight status and official failure differed between male and female students. Because official failure is based on the BMI-containing total score, these estimates were interpreted as national-standard surveillance findings rather than BMI-independent motor-fitness associations.

**Table 11:** Sex-stratified adjusted odds ratios and adjusted prevalence ratios for official failure of China’s national student physical health standard.

Predictor	Male adjusted OR (95% CI)	Male aPR (95% CI)	Female adjusted OR (95% CI)	Female aPR (95% CI)
Admission cohort 2024 vs. 2023	0.87 (0.72–1.04)	0.90 (0.80–1.01)	0.48 (0.35–0.65)	0.57 (0.45–0.72)
Admission cohort 2025 vs. 2023	2.29 (1.94–2.71)	1.54 (1.41–1.68)	1.28 (0.98–1.68)	1.17 (0.98–1.41)
Admission cohort 2025 vs. 2024	2.65 (2.22–3.17)	1.72 (1.55–1.91)	2.68 (1.96–3.65)	2.07 (1.63–2.63)
Underweight vs. normal weight	2.69 (2.09–3.46)	2.22 (1.82–2.69)	1.36 (0.85–2.18)	1.28 (0.87–1.88)
Overweight vs. normal weight	3.82 (3.18–4.57)	2.88 (2.51–3.31)	2.27 (1.68–3.05)	1.89 (1.51–2.36)
Obese vs. normal weight	18.17 (15.05–21.94)	6.09 (5.40–6.87)	7.65 (5.47–10.69)	3.75 (3.10–4.54)

Adjusted OR, odds ratio; aPR, adjusted prevalence ratio; CI, confidence interval. Sex-stratified models were fitted separately for male and female students. ORs were estimated using logistic regression, and aPRs were estimated using modified Poisson regression with a log link and robust sandwich standard errors. Models were adjusted for age, admission cohort, and weight status. Age was entered as a continuous covariate in decimal years but is not displayed as a focal predictor. Normal weight and the 2023 admission cohort were used as reference categories. The 2025-versus-2024 comparisons were obtained by refitting the models with 2024 as the reference admission cohort.

To provide absolute probability estimates, adjusted predicted probabilities of official failure were also estimated and are presented in Table 12.

**Table 12:** Adjusted predicted probabilities of official failure by admission cohort, weight status, and sex.

Factor	Category	Adjusted predicted probability of official failure
Admission cohort	2023	22.8%
Admission cohort	2024	18.7%
Admission cohort	2025	33.4%
Weight status	Normal weight	11.9%
Weight status	Underweight	22.4%
Weight status	Overweight	29.5%
Weight status	Obese	63.6%
Sex	Male	25.3%
Sex	Female	24.7%

Adjusted predicted probabilities were estimated using marginal standardization from the adjusted logistic regression model. Admission-cohort probabilities were adjusted for age, sex, and weight status. Weight-status probabilities were adjusted for age, sex, and admission cohort. Sex probabilities were adjusted for age, admission cohort, and weight status. Age was entered as a continuous covariate in decimal years.

Adjusted predicted probabilities provided absolute estimates that were easier to interpret than odds ratios alone. After adjustment for age, sex, and weight status, the predicted probability of official failure was 22.8% in the 2023 admission cohort, 18.7% in the 2024 admission cohort, and 33.4% in the 2025 admission cohort. After adjustment for age, sex, and admission cohort, the predicted probability was 11.9% among normal-weight students, 22.4% among underweight students, 29.5% among overweight students, and 63.6% among students with obesity. After adjustment for age, admission cohort, and weight status, the predicted probability of official failure was similar between male and female students, at 25.3 and 24.7%, respectively. These probability-based estimates followed the same pattern as the odds-ratio and adjusted prevalence-ratio models but provided a more directly interpretable measure of absolute failure probability. The higher adjusted probability of official failure in the 2025 cohort persisted after adjustment for age, sex, and weight status. However, these estimates should still be interpreted as within-institution cohort comparisons because other unmeasured cohort-composition factors, including vocational major, admission pathway, prior physical activity, and testing context, were unavailable.

To assess whether the weight-status associations persisted after removal of the BMI scoring component, BMI-free low motor-fitness performance was analysed as a binary sensitivity outcome using models adjusted for age, sex, and admission cohort. The comparison between official failure and BMI-free low motor-fitness performance is presented in Table 13.

**Table 13:** Adjusted sensitivity analysis comparing official failure and BMI-free low motor-fitness performance by weight status.

Weight status	Official failure OR (95% CI)	Official failure aPR (95% CI)	BMI-free low motor-fitness OR (95% CI)	BMI-free low motor-fitness aPR (95% CI)
Normal weight	Reference	Reference	Reference	Reference
Underweight	2.20 (1.77–2.74)	1.89 (1.59–2.24)	1.06 (0.89–1.27)	1.04 (0.94–1.15)
Overweight	3.24 (2.79–3.78)	2.52 (2.25–2.82)	1.75 (1.55–1.98)	1.35 (1.27–1.44)
Obese	14.49 (12.34–17.01)	5.31 (4.82–5.85)	2.86 (2.49–3.28)	1.64 (1.55–1.74)

OR, odds ratio; aPR, adjusted prevalence ratio; CI, confidence interval. ORs were estimated using logistic regression, and aPRs were estimated using modified Poisson regression with a log link and robust sandwich standard errors. All models were adjusted for age, sex, and admission cohort. Age was entered as a continuous covariate in decimal years. Normal weight was used as the reference category. Official failure refers to failure based on the BMI-containing official total score. BMI-free low motor-fitness performance was defined as a BMI-free motor-fitness score below 60.0 points and was used only as a sensitivity outcome.

**Figure 5:**
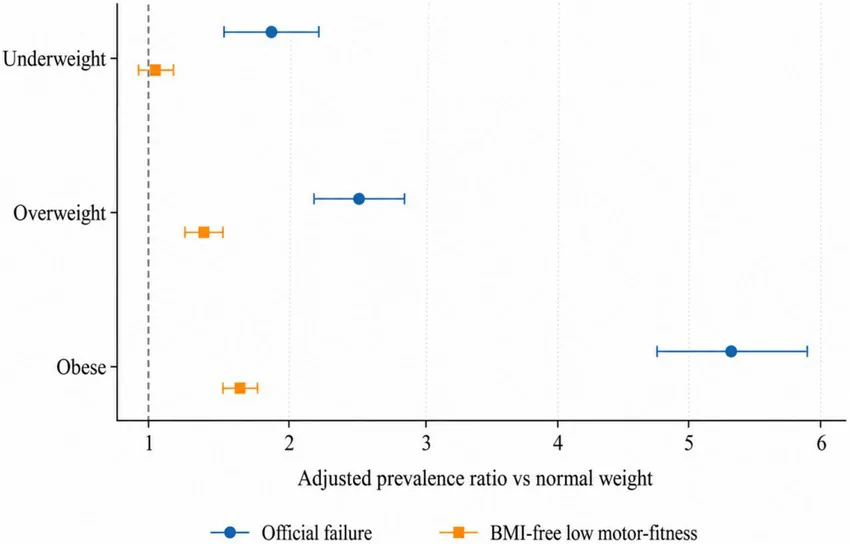
Adjusted prevalence ratios for official failure and BMI-free low motor-fitness performance by weight status. Adjusted prevalence ratios are shown relative to normal-weight students. Points indicate adjusted prevalence ratios, and horizontal bars indicate 95% confidence intervals. Models were adjusted for age, sex, and admission cohort. Official failure refers to failure based on the BMI-containing official total score. BMI-free low motor-fitness performance was defined as a BMI-free motor-fitness score below 60.0 points and was used only as a sensitivity outcome. The vertical dashed line indicates *aPR* = 1.00. Weight-status associations, particularly the obesity association, were substantially attenuated after removal of the BMI component but remained evident for overweight and obesity.

Compared with the corresponding odds ratios, adjusted prevalence ratios were smaller for both binary outcomes, as expected when the outcomes are common. For example, the obesity association decreased from an OR of 14.49 to an aPR of 5.31 for official failure and from an OR of 2.86 to an aPR of 1.64 for BMI-free low motor-fitness performance. Nevertheless, the overall interpretation remained unchanged after multivariable adjustment for age, sex, and admission cohort: overweight and obesity were associated with higher prevalence of both official failure and BMI-free low motor-fitness performance, although the BMI-free estimates were substantially attenuated after removal of the BMI component. For underweight students, the association was evident for official failure but was not clearly present for BMI-free low motor-fitness performance because the corresponding confidence intervals included the null. These findings further demonstrate that the very large association between obesity and official national-standard failure was partly attributable to the BMI component of the official scoring system, while lower performance-based motor fitness remained evident among students with overweight and obesity after BMI removal. The attenuation of the adjusted weight-status associations after removal of the BMI component is illustrated in Figure 5.

Multiple-comparison sensitivity analyses for the main adjusted logistic regression contrasts are presented in Table 14. The principal admission-cohort and weight-status associations remained statistically significant after both false discovery rate and Bonferroni correction.

**Table 14:** Multiple-comparison sensitivity analysis for key adjusted logistic regression contrasts.

Contrast	Raw *p*-value	FDR-adjusted *p*-value	Bonferroni-adjusted *p*-value
Admission cohort 2024 vs. 2023	<0.001	<0.001	<0.001
Admission cohort 2025 vs. 2023	<0.001	<0.001	<0.001
Admission cohort 2025 vs. 2024	<0.001	<0.001	<0.001
Underweight vs. normal weight	<0.001	<0.001	<0.001
Overweight vs. normal weight	<0.001	<0.001	<0.001
Obese vs. normal weight	<0.001	<0.001	<0.001

FDR, false discovery rate. Raw *p*-values were obtained from logistic regression models adjusted for age, sex, admission cohort, and weight status. Age was entered as a continuous covariate in decimal years. FDR-adjusted *p*-values were calculated using the Benjamini–Hochberg procedure. Bonferroni-adjusted *p*-values were calculated across the six selected admission-cohort and weight-status contrasts. All raw and adjusted *p*-values were <0.001.

### 3.5 Component-level fitness failure patterns

Component-level failure patterns were examined separately for male and female students because the national standard applies different endurance and muscular strength/endurance tests by sex and because male students represented more than 70% of each admission cohort. Sex-stratified failure prevalence for the individual physical fitness components is presented in Table 15.

**Table 15:** Sex-stratified failure prevalence of individual physical fitness components by admission cohort.

Fitness component	Male students			Female students		
	2023 (%)	2024 (%)	2025 (%)	2023 (%)	2024 (%)	2025 (%)
Vital capacity	5.5	4.7	3.5	2.3	1.9	2.0
50 m sprint	6.8	8.3	8.5	29.5	21.9	21.7
Standing long jump	28.9	26.7	28.9	29.5	29.3	26.6
Sit-and-reach	12.8	7.9	11.0	8.8	3.7	8.4
1,000 m run	48.2	50.7	56.7	—	—	—
800 m run	—	—	—	48.6	37.9	40.7
Pull-ups	82.3	83.7	75.1	—	—	—
One-minute sit-ups	—	—	—	27.7	20.3	23.8

Failure was defined as a component score below 60 points according to China’s National Student Physical Health Standard. Male endurance and muscular strength/endurance were assessed using the 1,000 m run and pull-ups, respectively. Female endurance and muscular strength/endurance were assessed using the 800 m run and one-minute sit-ups, respectively. Percentages were calculated within each sex-specific admission cohort. “—” dashes indicate that the test was not applicable to that sex. Because different tests and sex-specific scoring criteria were applied, male and female percentages should be interpreted as descriptive patterns within each sex rather than as directly equivalent test-performance measures.

Sex-stratified analyses showed that the aggregate component-failure pattern masked substantial differences between male and female students. Among male students, pull-ups had the highest failure prevalence in every admission cohort, with rates of 82.3% in 2023, 83.7% in 2024, and 75.1% in 2025. Failure in the 1,000 m run was also common and was observed in 48.2, 50.7, and 56.7% of male students across the three respective cohorts. Among female students, failure in the 800 m run was the most frequent component-level failure, with rates of 48.6% in 2023, 37.9% in 2024, and 40.7% in 2025. Failure in one-minute sit-ups was substantially lower than male pull-up failure, at 27.7, 20.3, and 23.8% across the three respective cohorts.

Sex-specific patterns were also evident for the 50 m sprint. Failure prevalence among female students was 29.5% in 2023, 21.9% in 2024, and 21.7% in 2025, compared with 6.8, 8.3, and 8.5% among male students. Standing-long-jump failure was comparatively similar between male and female students, while failure prevalence for vital capacity remained low in both groups. These findings demonstrate that the high aggregate muscular strength/endurance failure prevalence was driven largely by male pull-up performance and should not be interpreted as representing an equivalent pattern among female students. Endurance failure remained substantial in both sexes, although the cohort-specific patterns differed between the male 1,000 m run and female 800 m run.

In the combined sample, among students who failed the overall physical fitness standard, 92.8% failed the endurance component, 89.3% failed the muscular strength/endurance component, and 99.1% failed at least one of these two components. These aggregate percentages describe the overlap between component-level non-passing performance and overall official failure, but they should be interpreted alongside the sex-stratified findings because the relevant tests differed by sex and male students constituted the majority of the sample.

## 4 Discussion

### 4.1 Principal findings

This repeated cross-sectional study examined standardised physical fitness performance among three independent first-year admission cohorts at a single vocational college in Shanxi Province, China. Using China’s National Student Physical Health Standard and a large institutional dataset, the study provides contemporary within-institution surveillance evidence on cohort-level differences and weight-related disparities in physical fitness at entry into vocational education. Three principal findings emerged. First, although median official total physical fitness scores varied only modestly across cohorts, official failure prevalence differed across cohorts, with the lowest prevalence observed in 2024 and the highest prevalence observed in 2025. This divergence between relatively stable central tendency and higher official failure prevalence in 2025 suggests that fitness differences were not evenly distributed across the cohort; rather, a larger subgroup of students in 2025 appeared to fall near or below the minimum fitness threshold prescribed by the national standard.

Second, weight-status categories differed in official national-standard outcomes; however, these official-score associations must be interpreted in light of the inclusion of BMI in the national scoring framework. BMI-free motor-fitness sensitivity analyses showed that weight-related differences were attenuated after removal of the anthropometric scoring component, but lower performance-based motor fitness remained evident among students with overweight and obesity. Third, sex-stratified component analyses showed that endurance failure was substantial among both male and female students, whereas the high aggregate muscular strength/endurance failure prevalence was driven largely by male pull-up performance; failure in the female one-minute sit-up test was considerably lower. These findings identify aerobic capacity and sex-specific muscular strength/endurance as potential priorities for institutional fitness support.

### 4.2 Interpretation of admission-cohort differences in physical fitness

Official failure prevalence was 23.4% in the 2023 cohort, 17.0% in the 2024 cohort, and 34.8% in the 2025 cohort. These values compare independent first-year admission cohorts rather than changes within the same students. Because only three cohorts from one institution were included, the findings cannot establish a stable temporal trend, pandemic-related effect, or wider decline in the Chinese vocational-college population.

The distributional analyses provide a more precise interpretation of the higher failure prevalence in 2025. Median official total fitness scores differed only modestly between cohorts, and the adjusted median score in 2025 was not lower than that in 2024. In contrast, the 2025 distribution was wider and had lower scores at the 10th and 25th percentiles, indicating that the higher failure prevalence was concentrated primarily in the lower part of the distribution. The 2025 cohort therefore appeared to contain a larger subgroup near or below the passing threshold rather than showing a uniform reduction in fitness across all students.

This institutional pattern should be interpreted alongside, but not equated with, national and multi-year Chinese surveillance findings. Previous studies have identified long-term, regional, sex, grade-, and major-related variation in college-student fitness, while recent national evidence has also demonstrated regional inequalities and non-linear changes in fitness and BMI (8–12). The present results therefore contribute complementary local evidence rather than confirming a national temporal trajectory.

The available data cannot explain why the 2024 cohort had the lowest failure and overweight/obesity prevalence or why the 2025 cohort included a larger lower-performing subgroup. Although age was derived from date of birth and included in the adjusted analyses, information on admission pathway, vocational major and trade pathway, socioeconomic background, prior physical education and sport participation, habitual physical activity, sedentary behaviour, dietary habits, detailed day-to-day testing conditions, student motivation, and local implementation was unavailable. Differences in these factors could have influenced cohort composition, weight status, or physical fitness performance, but they were not measured and should not be treated as established explanations for the observed cohort differences. The findings therefore support the value of entry-level surveillance for identifying students close to the official failure threshold at the study institution, rather than conclusions about vocational-college students more broadly. Multi-institutional studies incorporating educational, behavioural, socioeconomic, programme-specific, and testing-context information are needed to determine whether similar lower-tail patterns occur in other vocational colleges and future admission cohorts.

### 4.3 Weight-status differences and the BMI component of the scoring system

A central methodological issue is that BMI serves both as the basis for weight-status classification and as a component of China’s National Student Physical Health Standard. Associations between weight status and the official total score or official failure are therefore not independent of the scoring structure. The BMI-free analyses addressed this circularity by removing the BMI component, rescaling the remaining performance-based components, and retaining recorded performance bonus points. Differences associated with overweight and obesity were attenuated but remained evident, indicating that the large official weight-status associations were partly attributable to BMI scoring but were not explained by it alone. This pattern is consistent with Chinese studies reporting lower endurance, speed, strength, power, or composite fitness at higher BMI or fat-mass levels (12, 18–20). Evidence concerning normal-weight obesity further indicates that students within the same BMI category may differ in fat mass, skeletal muscle mass, and physical fitness (21). The BMI-free outcome therefore reduces one source of structural circularity, but it does not substitute for direct assessment of body composition.

The adjusted prevalence ratios and predicted probabilities provided complementary interpretations of the binary outcomes. Because official failure was common, prevalence ratios were smaller and more directly interpretable as relative prevalence measures than the corresponding odds ratios, while predicted probabilities quantified absolute failure probability. The substantially higher adjusted probability of official failure among students with obesity demonstrates the practical burden of the official outcome but must still be interpreted partly within the BMI-containing scoring framework. Lower BMI-free performance among students with overweight and obesity may be consistent with the greater mechanical demands imposed by body mass during running, jumping, and body-weight-based strength tasks. Nevertheless, the observational design does not establish that body weight caused the performance differences. Physical activity, sedentary behaviour, diet, previous sport participation, socioeconomic circumstances, motivation, body composition, and testing context may influence both weight status and fitness.

The official and BMI-free outcomes should consequently be interpreted as two related but distinct layers of evidence. The official score indicates performance under the national surveillance and grading framework used by the institution, whereas the BMI-free score provides a more conservative assessment of performance-based motor fitness without the direct BMI contribution. The findings support interventions that address aerobic conditioning and muscular endurance rather than focusing exclusively on weight reduction. For students with overweight, obesity, or low baseline fitness, programmes should prioritise progressive participation, safety, confidence, and adherence.

### 4.4 Component-level fitness patterns and practical implications

Sex-stratified component analyses clarified that the aggregate component-failure rates masked different performance patterns among male and female students. Among male students, pull-ups had the highest failure prevalence in every cohort, while non-passing performance in the 1,000 m run was also common. Among female students, the 800 m run had the highest component-level failure prevalence, whereas failure in one-minute sit-ups was substantially lower than male pull-up failure. The high aggregate muscular strength/endurance failure prevalence therefore largely reflected the combination of the male-dominated sample and the high prevalence of non-passing pull-up performance among male students. Endurance failure remained substantial in both sexes, although the cohort-specific pattern differed between the male 1,000 m run and female 800 m run. The sex-specific results also showed higher 50 m sprint failure prevalence among female students than among male students, while standing-long-jump failure was comparatively similar. Because the national standard applies different tests and sex-specific scoring criteria, these results should be interpreted as within-sex patterns rather than as direct comparisons of identical performance tasks.

The sex-stratified findings also require contextual interpretation. Male students represented 70.38–74.76% of the three cohorts and had higher crude overweight/obesity and official failure prevalence, while the sex-by-weight-status interaction indicated that official weight-status associations differed between male and female students. Consequently, the aggregate estimates reflect the predominantly male composition of the study institution and may not apply to vocational colleges with different sex distributions or programme structures. However, vocational major and occupation-specific requirements were unavailable, and the national standard applies different tests to male and female students. The results should therefore not be used to make broad assumptions about occupational capability. Research among Chinese higher-vocational students has documented associations involving lifestyle and weight change, sleep and mental well-being, motivational support, and exercise adherence (13–15). These studies indicate that interventions in vocational settings should consider behavioural, motivational, and psychosocial context, although those factors were not measured in the present study.

At the study institution, entry-level screening may help identify students requiring additional support early in their programmes. Institution-specific physical-education support could include progressive aerobic conditioning and muscular-endurance training for students below or near the passing threshold, while any programme-related work-readiness activities should be developed only after considering the relevant vocational major and occupational requirements. Evidence from Chinese university settings suggests that structured physical-education interventions can improve physical-fitness outcomes, although comparable intervention trials in vocational colleges remain limited (23). For students with obesity or very low baseline fitness, adapted programmes should prioritise gradual progression, injury-risk reduction, confidence, and sustained adherence rather than immediate high-intensity exercise.

### 4.5 Strengths and limitations

This study has several strengths. The use of a large sample drawn from three consecutive admission cohorts enhances the stability of the descriptive and regression findings. The implementation of a nationally standardised physical fitness assessment framework supported consistency of measurement across years and facilitated comparison with studies conducted within the same system. In addition, the focus on first-year students assessed at entry into vocational college reduces potential confounding by later curricular exposure, institutional physical activity programmes, and changes occurring during vocational study.

Several limitations should also be considered. First, the repeated cross-sectional design precludes inference regarding individual-level changes in fitness over time, and causal relationships cannot be established. The design also cannot distinguish cohort effects from broader period effects. Because the study included only three independent first-year admission cohorts from one vocational college, the observed higher official failure prevalence in 2025 should not be interpreted as evidence of a pandemic-related effect, secular decline, or broader deterioration among vocational college students in China. Therefore, the discussion of possible explanations for the 2025 cohort should be read as contextual interpretation rather than causal evidence.

Second, although only three records were excluded because of incomplete anthropometric and component-level assessment data, selection bias arising from missing or incomplete records cannot be entirely ruled out. However, the excluded records represented only 0.04% of the initially available records, making material bias from incomplete records unlikely. Third, the study was conducted at a single vocational college in Shanxi Province and did not use population-based or multi-institutional sampling. The sample was predominantly male, with male students comprising 70.38–74.76% of the three admission cohorts. This sex distribution reflects the composition of the available institutional cohorts; because vocational-major data were unavailable, the contribution of programme mix could not be examined. Although sex-stratified descriptive and regression analyses were conducted, the aggregate prevalence estimates and associations may not apply to vocational colleges with different sex distributions, programme structures, admission pathways, or student populations. These sampling characteristics restrict the generalisability of the findings to other institutions, regions, vocational programmes, and the broader vocational-college student population in China. Fourth, weight status was classified using BMI, which does not differentiate between fat mass and lean mass and may result in misclassification, particularly in relation to strength-based fitness components. Because individual assessment dates were unavailable, age was calculated using a common reference date within each assessment year, which may have introduced minor measurement imprecision; however, the same method was applied consistently across all cohorts.

An additional limitation concerns the structure of the national scoring system. BMI is used both to classify weight status and as one component of the official total physical fitness score. Therefore, associations between weight status and official total score or official failure may partly reflect this built-in scoring structure. To reduce this concern, BMI-free motor-fitness sensitivity analyses were conducted by removing the BMI component and rescaling the remaining performance-based components. Nevertheless, the BMI-free score is a recalculated research outcome rather than an official national-standard score. It retained performance bonus points awarded under the national standard because these points reflected exceptional motor-fitness performance rather than BMI classification. The official failure outcome should therefore be interpreted as a surveillance indicator based on the full national scoring framework, while the BMI-free score should be interpreted only as a research-derived sensitivity outcome. Although odds ratios were retained for comparability, official failure and BMI-free low motor-fitness were common outcomes; therefore, adjusted prevalence ratios and adjusted predicted probabilities were added and should be emphasised when interpreting the magnitude of association.

Finally, several potentially important behavioural, educational, institutional, and social variables were unavailable. These included vocational major and trade pathway, socioeconomic background, physical activity, sedentary behaviour, dietary habits, prior sport participation, secondary-school physical education, admission pathway, student motivation, detailed day-to-day testing conditions, and local implementation. Although the formal testing window, assessment protocol, scoring criteria, and equipment were unchanged according to institutional records, more detailed testing-context information was not available for analysis. These unmeasured factors may be associated with cohort composition, weight status, and physical fitness and could therefore confound or partly explain the observed admission-cohort and weight-status differences. They should be regarded as limitations of interpretation rather than as explanations supported by the present data.

More specifically, the dataset did not contain information on parental education, household economic circumstances, family affluence, rural or urban origin, or previous access to organised physical activity. Chinese research has identified socioeconomic differences in physical activity among young people, associations between parental socioeconomic status and skeletal muscle mass among college students, and regional inequalities in college-student fitness and BMI (12, 16, 17). The absence of these variables limits the ability to determine whether social or educational inequalities contributed to the observed admission-cohort or weight-status differences. Information on students’ vocational majors and trade pathways was also unavailable, preventing direct assessment of programme-specific fitness requirements.

*E*-values provide a supplementary indication of the magnitude of unmeasured confounding required to move an association to the null but do not address measurement error, selection bias, model misspecification, causality, or the structural overlap between BMI classification and the BMI-containing official outcome. Multiple-comparison sensitivity analyses supported the principal admission-cohort and weight-status associations, although secondary and exploratory comparisons should still be interpreted cautiously.

## 5 Conclusion

This repeated cross-sectional study identified differences in official national-standard physical fitness outcomes across three independent first-year admission cohorts at a single vocational college in Shanxi Province, China. Median official total fitness scores varied only modestly across cohorts, but official failure prevalence was lowest in 2024 and highest in 2025. Distributional analyses indicated that the higher failure prevalence in 2025 reflected a larger lower-performing subgroup near or below the passing threshold rather than a uniform decline in fitness across the cohort. These findings represent within-institution differences between independent admission cohorts and do not establish individual-level or broader population-level temporal change.

Weight status was associated with official total-score and failure outcomes, although these associations were partly influenced by the inclusion of BMI in the official scoring framework. BMI-free sensitivity analyses attenuated the observed differences but showed that students with overweight or obesity continued to have lower performance-based motor fitness. Sex-stratified component analyses showed that endurance failure was substantial in both sexes, while the high aggregate muscular strength/endurance failure prevalence was driven largely by male pull-up performance. Continued entry-level surveillance, together with progressively structured aerobic and muscular-endurance support, may help the study institution identify and support students at greater risk of low fitness performance. Because the sample was drawn from a single, predominantly male vocational college in Shanxi Province, the reported prevalence estimates and associations should not be interpreted as representative of first-year vocational college students across China. Multi-institutional studies with broader age and sex distributions and detailed information on vocational major, socioeconomic background, physical activity, sedentary behaviour, dietary habits, prior sport participation, body composition, and testing conditions are needed to determine whether similar patterns occur in other vocational-college populations.

## Data Availability

The original contributions presented in the study are included in the article/Supplementary material, further inquiries can be directed to the corresponding author.
