# Supplementary material for: Admission-cohort differences in physical fitness, weight status, and fitness-standard failure among first-year students at a single vocational college in Shanxi Province, China
Source: Front Public Health. 2026 Aug 10;14:1850051. doi: 10.3389/fpubh.2026.1850051 (PMC13500571; doi:10.3389/fpubh.2026.1850051)
Supplement: Supplementary file 1 [file Data_Sheet_1.PDF]

Supplementary File 1.

Scoring criteria used for China's National Student Physical Health Standard

The following tables summarise the scoring criteria used in this study based on China's National Student Physical Health Standard issued by the Ministry of Education of China in 2014. The original criteria were published in Chinese and are presented here in English for transparency and reproducibility. For the present study, all participants were first-year students; therefore, the Year 1–2 college student criteria were applied in the analysis.

Table S1 - Weight of each component in the total physical fitness score

Component	Weight (%)
BMI	15
Vital capacity	15
50 m sprint	20
Sit-and-reach	10
Standing long jump	10
Pull-ups (male) / 1 min sit-ups (female)	10
1000 m run (male) / 800 m run (female)	20

Table S2 - Overall total-score grading criteria

Total score	Grade
≥ 90.0	Excellent
80.0–89.9	Good
60.0–79.9	Pass
≤ 59.9	Fail

Table S3 - BMI classification criteria

Weight status	Score	Male BMI (kg/m²)	Female BMI (kg/m²)
Normal weight	100	17.9–23.9	17.2–23.9
Underweight	80	≤ 17.8	≤ 17.1
Overweight	80	24.0–27.9	24.0–27.9
Obese	60	≥ 28.0	≥ 28.0

Table S4 - Vital capacity scoring table

Score	Male Y1–2	Male Y3–4	Female Y1–2	Female Y3–4
100	5040	5140	3400	3450
95	4920	5020	3350	3400
90	4800	4900	3300	3350

85	4550	4650	3150	3200
80	4300	4400	3000	3050
78	4180	4280	2900	2950
76	4060	4160	2800	2850
74	3940	4040	2700	2750
72	3820	3920	2600	2650
70	3700	3800	2500	2550
68	3580	3680	2400	2450
66	3460	3560	2300	2350
64	3340	3440	2200	2250
62	3220	3320	2100	2150
60	3100	3200	2000	2050
50	2940	3030	1960	2010
40	2780	2860	1920	1970
30	2620	2690	1880	1930
20	2460	2520	1840	1890
10	2300	2350	1800	1850

Table S5 - 50 m sprint scoring table

Score	Male Y1–2	Male Y3–4	Female Y1–2	Female Y3–4
100	6.7	6.6	7.5	7.4
95	6.8	6.7	7.6	7.5
90	6.9	6.8	7.7	7.6
85	7.0	6.9	8.0	7.9
80	7.1	7.0	8.3	8.2
78	7.3	7.2	8.5	8.4
76	7.5	7.4	8.7	8.6
74	7.7	7.6	8.9	8.8
72	7.9	7.8	9.1	9.0
70	8.1	8.0	9.3	9.2
68	8.3	8.2	9.5	9.4
66	8.5	8.4	9.7	9.6
64	8.7	8.6	9.9	9.8
62	8.9	8.8	10.1	10.0
60	9.1	9.0	10.3	10.2
50	9.3	9.2	10.5	10.4

40	9.5	9.4	10.7	10.6
30	9.7	9.6	10.9	10.8
20	9.9	9.8	11.1	11.0
10	10.1	10.0	11.3	11.2

Table S6 - Sit-and-reach scoring table

Score	Male Y1–2	Male Y3–4	Female Y1–2	Female Y3–4
100	24.9	25.1	25.8	26.3
95	23.1	23.3	24.0	24.4
90	21.3	21.5	22.2	22.4
85	19.5	19.9	20.6	21.0
80	17.7	18.2	19.0	19.5
78	16.3	16.8	17.7	18.2
76	14.9	15.4	16.4	16.9
74	13.5	14.0	15.1	15.6
72	12.1	12.6	13.8	14.3
70	10.7	11.2	12.5	13.0
68	9.3	9.8	11.2	11.7
66	7.9	8.4	9.9	10.4
64	6.5	7.0	8.6	9.1
62	5.1	5.6	7.3	7.8
60	3.7	4.2	6.0	6.5
50	2.7	3.2	5.2	5.7
40	1.7	2.2	4.4	4.9
30	0.7	1.2	3.6	4.1
20	-0.3	0.2	2.8	3.3
10	-1.3	-0.8	2.0	2.5

Table S7 - Standing long jump scoring table

Score	Male Y1–2	Male Y3–4	Female Y1–2	Female Y3–4
100	273	275	207	208
95	268	270	201	202
90	263	265	195	196
85	256	258	188	189
80	248	250	181	182
78	244	246	178	179
76	240	242	175	176

74	236	238	172	173
72	232	234	169	170
70	228	230	166	167
68	224	226	163	164
66	220	222	160	161
64	216	218	157	158
62	212	214	154	155
60	208	210	151	152
50	203	205	146	147
40	198	200	141	142
30	193	195	136	137
20	188	190	131	132
10	183	185	126	127

Table S8 - Pull-ups / one-minute sit-ups scoring table

Score	Male Y1–2	Male Y3–4
100	19	20
95	18	19
90	17	18
85	16	17
80	15	16
76	14	15
72	13	14
68	12	13
64	11	12
60	10	11
50	9	10
40	8	9
30	7	8
20	6	7
10	5	6

Score	Female Y1–2	Female Y3–4
100	56	57
95	54	55
90	52	53

85	49	50
80	46	47
78	44	45
76	42	43
74	40	41
72	38	39
70	36	37
68	34	35
66	32	33
64	30	31
62	28	29
60	26	27
50	24	25
40	22	23
30	20	21
20	18	19
10	16	17

Table S9 - 1000 m / 800 m endurance run scoring table

Score	Male 1000 m Y1–2	Male 1000 m Y3–4	Female 800 m Y1–2	Female 800 m Y3–4
100	3'17"	3'15"	3'18"	3'16"
95	3'22"	3'20"	3'24"	3'22"
90	3'27"	3'25"	3'30"	3'28"
85	3'34"	3'32"	3'37"	3'35"
80	3'42"	3'40"	3'44"	3'42"
78	3'47"	3'45"	3'49"	3'47"
76	3'52"	3'50"	3'54"	3'52"
74	3'57"	3'55"	3'59"	3'57"
72	4'02"	4'00"	4'04"	4'02"
70	4'07"	4'05"	4'09"	4'07"
68	4'12"	4'10"	4'14"	4'12"
66	4'17"	4'15"	4'19"	4'17"
64	4'22"	4'20"	4'24"	4'22"
62	4'27"	4'25"	4'29"	4'27"
60	4'32"	4'30"	4'34"	4'32"
50	4'52"	4'50"	4'44"	4'42"

40	5'12"	5'10"	4'54"	4'52"
30	5'32"	5'30"	5'04"	5'02"
20	5'52"	5'50"	5'14"	5'12"
10	6'12"	6'10"	5'24"	5'22"

Table S10 - Bonus scoring criteria for pull-ups and one-minute sit-ups

Bonus points	Male pull-ups Y1–2	Male pull-ups Y3–4	Female sit-ups Y1–2	Female sit-ups Y3–4
10	10	10	13	13
9	9	9	12	12
8	8	8	11	11
7	7	7	10	10
6	6	6	9	9
5	5	5	8	8
4	4	4	7	7
3	3	3	6	6
2	2	2	4	4
1	1	1	2	2

Table S11 - Bonus scoring criteria for 1000 m / 800 m endurance run

Bonus points	Male 1000 m Y1–2	Male 1000 m Y3–4	Female 800 m Y1–2	Female 800 m Y3–4
10	-35"	-35"	-50"	-50"
9	-32"	-32"	-45"	-45"
8	-29"	-29"	-40"	-40"
7	-26"	-26"	-35"	-35"
6	-23"	-23"	-30"	-30"
5	-20"	-20"	-25"	-25"
4	-16"	-16"	-20"	-20"
3	-12"	-12"	-15"	-15"
2	-8"	-8"	-10"	-10"
1	-4"	-4"	-5"	-5"

Note: For the present analysis, component-level failure was defined as a component score below 60 points after raw performance values were converted according to the sex-specific scoring tables. Male students completed the 1000 m run and pull-ups, while female students completed the 800 m run and one-minute sit-ups. For time-based tests, lower values indicate better performance; for distance-, capacity-, and repetition-based tests, higher values indicate better performance.

Source: China’s National Student Physical Health Standard, Ministry of Education of China, 2014.
